# The neuroprotective role of CncC in a *Drosophila* model of Parkinson’s disease

**DOI:** 10.1371/journal.pone.0322640

**Published:** 2025-05-13

**Authors:** Terence Al L. Abaquita, Milena Damulewicz, Elżbieta Pyza

**Affiliations:** Department of Cell Biology and Imaging, Institute of Zoology and Biomedical Research, Jagiellonian University, Cracow, Poland; Baylor College of Medicine, UNITED STATES OF AMERICA

## Abstract

Parkinson’s disease (PD) is an incurable neurodegenerative disorder, yet significant advancements have been made in understanding its etiology. Among the risk factors, exposure to neurotoxins plays the greatest role. One of the most dangerous toxins is rotenone, a naturally derived compound that was historically used as an insecticide. This chemical affects mitochondrial function by blocking electron transfer, resulting in increased reactive oxygen species production and accumulation. Recently, the role of the Nrf2 pathway was explored as a possible protective mechanism to minimize the neurotoxic effects leading to Parkinson’s disease. Here, we used *Drosophila melanogaster* as a model to examine the link between the expression or activity levels of CncC (an ortholog of *Nrf2*) or HO (an ortholog of *HO-1*) in the brain and the detrimental effects of chronic exposure to rotenone. We found that flies with overexpression of *CncC* or silencing of *ho* survived better after exposure to rotenone compared with flies with partially suppressed *CncC* or upregulated *ho* expression. These experimental groups exposed to rotenone also exhibited significantly fewer degenerated dopaminergic (DA) neurons than did the wild-type group. Nevertheless, only those in which *CncC* was overexpressed in glia showed the best survival, the greatest percentage of climbing ability, and no effects on DA neurons. Our findings were supported by data obtained for flies fed with HO inhibitor (SnPPIX) or activator (hemin), as well as with curcumin (Nrf2 activator). The observed effects were connected with changes in autophagy and apoptosis pathways. Our data suggest that possible therapies exploiting Nrf2 activation should include restricting HO upregulation as a neuroprotective strategy against the toxic effects of rotenone.

## Introduction

Parkinson’s disease (PD) is one of the most common neurodegenerative diseases [1–3] and is characterized by tremors, rigidity, slowness of movement, and postural instability [4–6]. Despite major advances in understanding the etiology of PD, its mechanisms remain elusive and multifactorial. Accordingly, approximately 95% of Parkinsonism cases are considered sporadic [7] and are known to be induced by both environmental and genetic factors [8–12].

Both clinical and experimental studies have shown that defective mitochondrial function and increased oxidative stress play a central role in the pathogenesis of PD [7,13–16]. Both conditions can regulate and activate cell death pathways [17,18], leading to neuronal degeneration [19,20]. The most common neurotoxin used to induce sporadic PD in animal models is rotenone [21]. It has been shown *in vivo* that after chronic exposure to rotenone, both oxidative injury and mitochondrial dysfunction are observed, leading to cluster-specific dopaminergic (DA) neuron loss and motor deficits [22–25]. In addition, cytoplasmic inclusions similar to Lewy bodies [26] and the accumulation of iron and ubiquitin [27] were also observed. In total, rotenone stimulates oxidative damage, endoplasmic reticulum stress, autophagy flux inhibition, lysosomal dysfunction, and cell death [28–31].

It has been suggested that nuclear factor erythroid 2-related factor 2 (Nrf2) serves as a vital defense against oxidative stress and mitochondrial impairment [32]. Once freed from Kelch ECH-associated protein 1 (Keap1) during oxidative stress, the stabilized transcription factor Nrf2 is transferred from the cytoplasm to the nucleus. Then, it binds to the antioxidant response element (ARE) to activate the expression of cytoprotective and antioxidative enzymes, including heme oxygenase-1 (HO-1) [33–37]. As a consequence, it improves mitochondrial function and ATP synthesis and prevents oxidative damage [38]. Another important role of Nrf2 is its involvement in the regulation of mitochondrial biogenesis and dynamics as well as mitophagy [38]. Mitophagy is an autophagic process that eliminates damaged, old, or improperly functioning mitochondria [39], and upregulation of Nrf2 can be a potential strategy for reducing the oxidative damage and mitochondrial failure present in PD.

In vertebrates, Nrf2 belongs to the Cap ‘n’ Collar (CNC) bZIP family of transcription factors and shares high homology with CncC present in *Drosophila melanogaster* [36]. Like mammalian Nrf2, CncC in *Drosophila* controls growth, aging, stress-responsive gene expression and regulates proteasomes. In contrast, the fly CncC has been demonstrated to regulate the expression of genes involved in all phases of detoxification, including resistance to pesticides [37]. The HO-1 homolog has also been described in *Drosophila* (dHO), which is presumed to be under the regulation of CncC. dHO is encoded by a single gene (*ho*) and degrades heme to biliverdin, carbon monoxide (CO), and ferrous ions [40]. Several processes that are indispensable for cellular homeostasis, such as iron accumulation [41], DNA damage signaling [42], protection against DNA damage caused by UV and white light [43,44], DNA repair, immune responses [45], circadian clocks [43,45,46], apoptosis [41,47,48] and autophagy [47,48], have been revealed to be under the control of *ho*. Pro-oxidative conditions (i.e., aging, heat stress, and paraquat) were reported to decrease *ho* expression in the brain. However, in the case of paraquat, *ho* was shown to have a time-dependent response, and its expression increased at the time when the flies exhibited high locomotor activity [47]. Changes in *ho* expression have been shown to influence normal development, survival, fitness, and sleep [41,49]. Additionally, it has been suggested that disturbances in proper HO-1 and *ho* mRNA levels are associated with neurodegeneration [48,50–55].

In the present study, we aimed to further explore the neuroprotective mechanisms of the HO and Nrf2 pathways against oxidative damage and mitochondrial failure. To achieve this goal, we used an experimental approach, in which *Drosophila* were exposed to rotenone to produce PD-like phenotypes [56]. Flies with modified expression of *ho* or *CncC* in neurons or in glia (through the GAL4/UAS system) were subjected to rotenone-induced effects. The results showed that the upregulation of *CncC* could be a protective intervention against neurodegeneration induced by environmental toxins.

## Results

### Rotenone exposure affects heme oxygenase, autophagy, and apoptosis gene expression in fly heads

**Fig 1:**
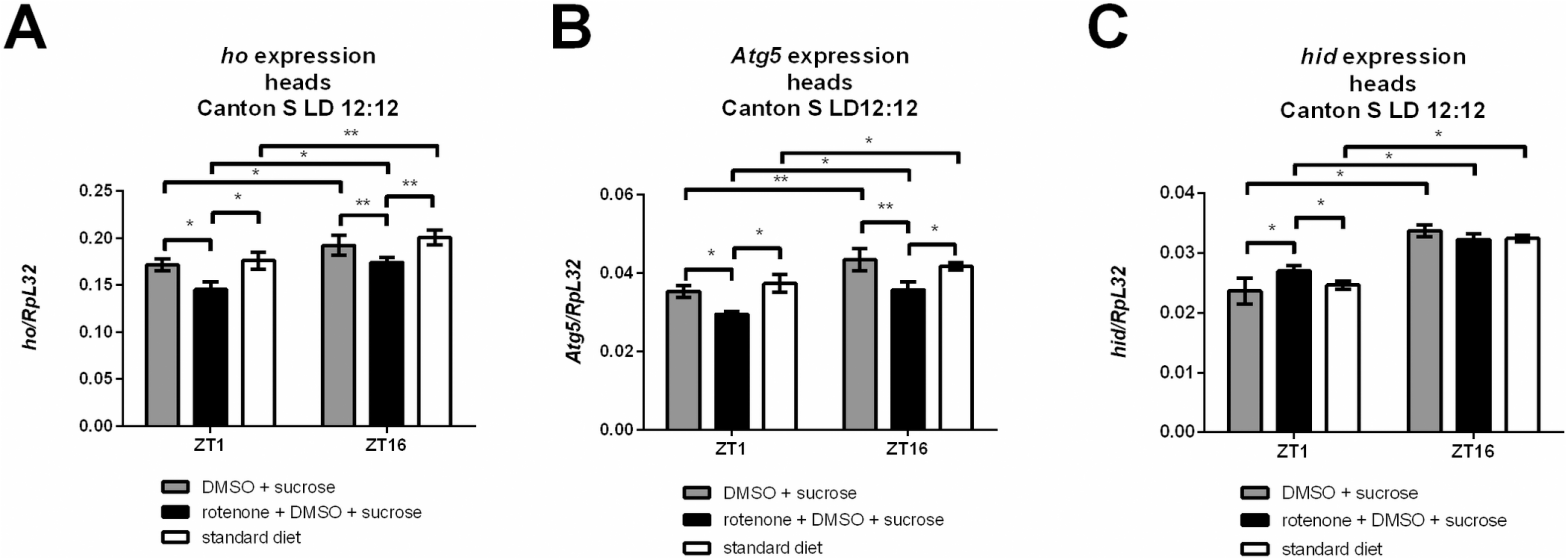
Gene expression after rotenone exposure. Wild-type flies (2 days old) were fed with rotenone for 7 d. Changes in the expression of heme oxygenase (*ho*, A), autophagy gene (*Atg5*, B) and apoptotic gene (*hid*, C) were detected compared with those in the controls. Mean ± SEM. Statistically significant changes are marked with asterisks: *p < 0.05, **p < 0.01.

To check the effect of rotenone treatment on the selected gene expression we fed flies with this neurotoxin dissolved in DMSO and 10% sucrose solution. To exclude the potential effect of DMSO (used as a solvent) on gene expression, we compared the experimental group of flies with two control groups — one fed a standard diet and the other fed with DMSO and sucrose. We did not observe any effect of DMSO on the expression levels of the studied genes (Fig 1). Since many genes have different expression levels during the day, we collected all samples strictly at two time points: at the beginning of the day (ZT1) and during the night (ZT16). These time points were selected based on the previous study, in which we showed that *ho* expression in the brain is rhythmic and reaches the highest levels at ZT1 and ZT16. The results showed that after rotenone treatment, the *ho* mRNA level decreased at both time points compared with that in the controls (Fig 1A). Similar changes were observed for the selected autophagy-related gene *Atg5* (Fig 1B). Finally, we examined the expression of *head involution defective* (*hid)*, a marker of apoptosis, and found that the expression of this gene increased at ZT1 but was not affected at ZT16 (Fig 1C).

### Changes in the expression levels of *ho* or *CncC* in the brain influence adult longevity and climbing behavior after rotenone exposure

**Fig 2:**
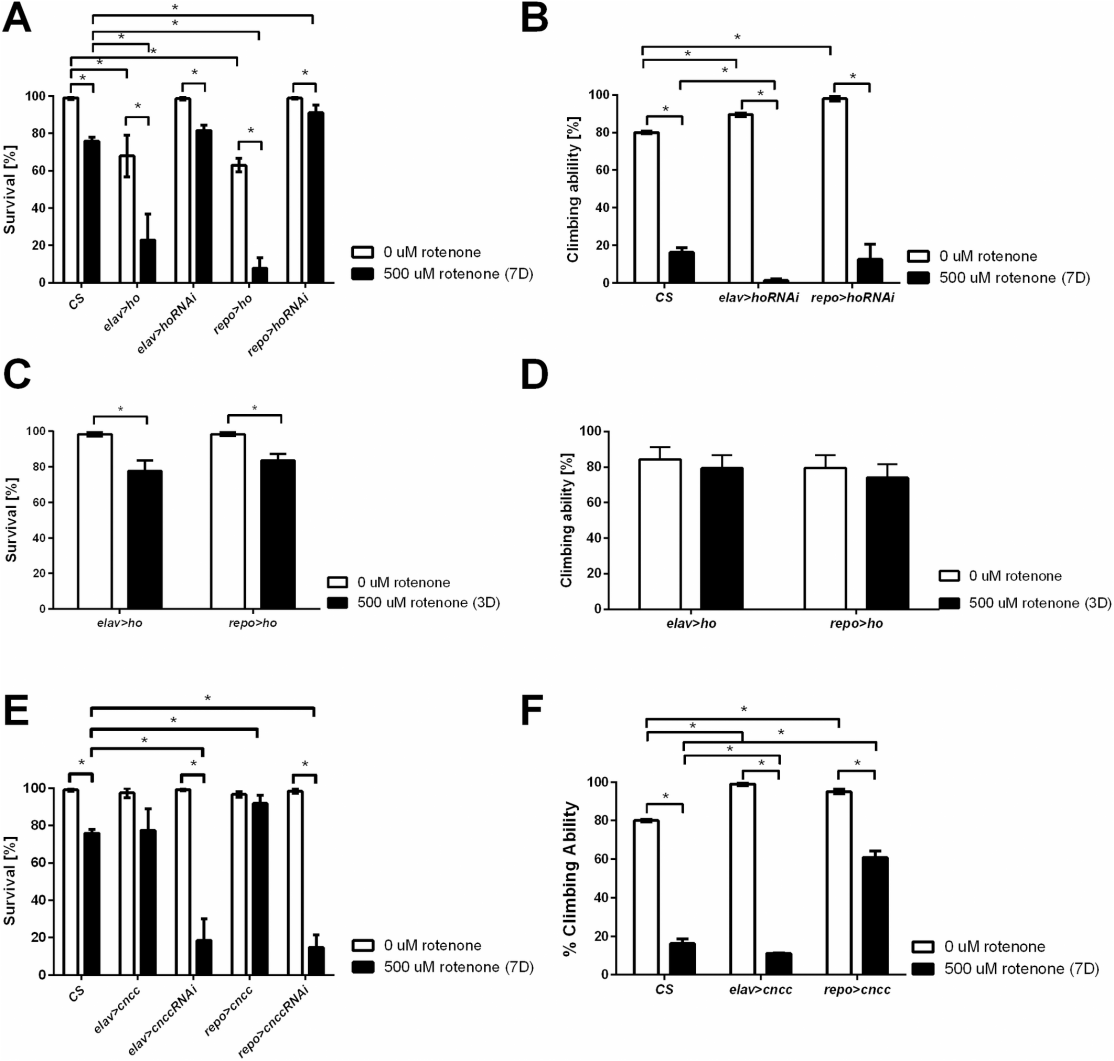
Effects of *ho* and *CncC* overexpression and silencing in neurons (*elav*) and glia (*repo*) on the survival and climbing ability of flies treated with rotenone for 7 days (A, B, E, F) or 3 days (C, D), respectively. Mean ± SEM. Statistically significant differences are marked with asterisks; *p < 0.05.

Rotenone is a strong neurotoxin, and as expected, the survival of wild-type flies (CantonS, CS) after 7 days of exposure decreased by 20% (Fig 2A). Surprisingly, overexpression of *ho*, both in neurons and glia, strongly reduced the percentage of surviving flies to 10–15% after rotenone treatment. On the other hand, approximately 80% of the flies with pan-neuronal *ho* silencing survived exposure to rotenone, and this effect was similar to that of the wild-type flies, while the *repo*>*hoRNAi* flies (pan-glial silencing) showed significantly greater survival than the wild-type flies (Fig 2A). Despite a high percentage of surviving flies, rotenone caused climbing defects in flies with decreased *ho* expression levels in neurons in comparison to those that were not exposed (≥ 85% difference between treated and nontreated with rotenone), which was greater than that observed in CS flies (65%) (Fig 2B). Glial *ho*-silenced flies treated with rotenone behaved similar to wild-type flies under the same conditions (Fig 2B). We were not able to examine climbing ability in flies with *ho* overexpression after 7 days of rotenone exposure (*elav*>*ho* or *repo*>*ho*) because only a few flies survived until the end of the experiment. In this part, we carried out experiments only after 3 days of rotenone exposure and observed that the survival of the flies was approximately 80% (Fig 2C) (similar to that of the CS after 7 days of rotenone), while climbing was not affected (Fig 2D).

Interestingly, partial silencing of *CncC* in neurons or glia decreased the tolerance of the experimental flies to chronic rotenone exposure and decreased survival compared with that of the wild-type flies (Fig 2E). Surprisingly, *CncC* overexpression in glia (*repo*>*CncC*) did not affect survival in flies exposed to rotenone (Fig 2E). Accordingly, compared with control flies, those with neurons overexpressing *CncC* exhibited similar changes in climbing ability after rotenone treatment (Fig 2F). However, compared to wild-type flies, *repo*>*CncC* treated flies exhibited better climbing performance (34% difference between treated and untreated flies) (Fig 2F). Climbing assays were not carried out with *elav*>*CncCRNAi* and *repo*>*CncCRNAi* flies because of their very low survival. This was also the reason to discontinue experiments with flies in which *CncC* was silenced.

To check whether observed effects were connected with the oxidative stress we compared ROS levels in heads of flies treated and untreated with rotenone (Fig S1). In *elav>CncC* flies treated with rotenone ROS level showed 171,3% of control and in *repo>CncC* reached 516,4%, however because the basal level of ROS in *repo>CncC* flies was very low, after rotenone feeding it reached the same level as in *elav>CncC* flies. *ho* overexpression extremely increased oxidative stress (*elav>ho* 507,7%, *repo>ho* 882%), while flies with *ho* silencing showed lower changes in ROS level after rotenone treatment (*elav>hoRNAi* 162,4% and *repo>hoRNAi* 229,3%).

### The effect of rotenone on gene expression depends on HO and CncC levels in the brain

**Fig 3:**
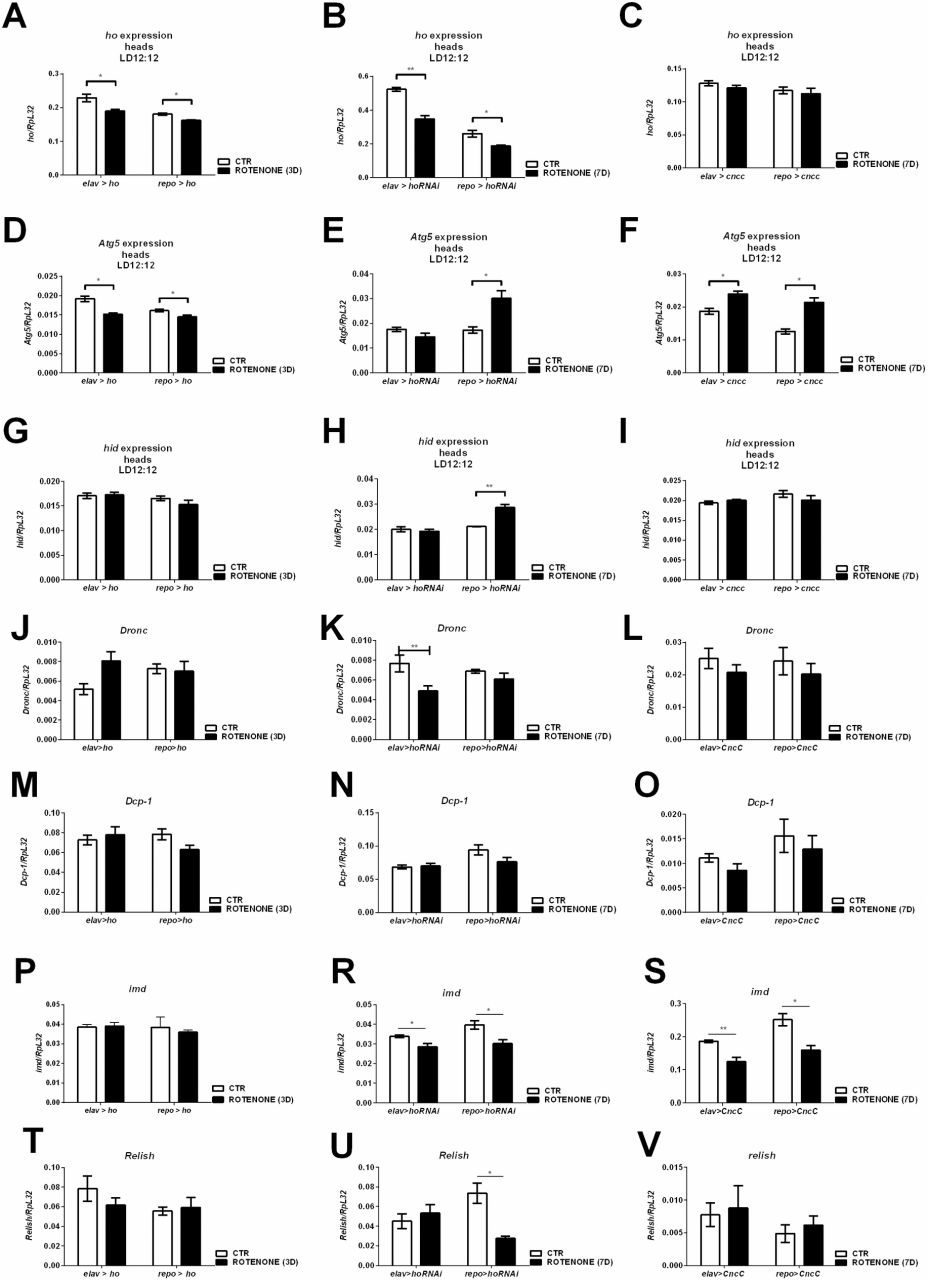
Gene expression in flies with *ho* and *CncC* overexpression or silencing in neurons or glia after 3 or 7 days of rotenone treatment. Mean ± SEM. Statistically significant differences are marked with asterisks; *p < 0.05, **p < 0.01.

To explain the changes observed in survival and climbing after rotenone treatment, we measured the expression levels of selected genes in the heads of the experimental flies. In flies with *ho* overexpression in neurons or glia, after 3 days of rotenone feeding, the *ho* mRNA level decreased (Fig 3A). A similar effect was observed for the *elav*>*hoRNAi* and *repo*>*hoRNAi* strains after 7 days of treatment, when the level of *ho* mRNA significantly decreased (Fig 3B). Surprisingly, in both neurons and glia of flies with *CncC* overexpression, the *ho* expression level was not affected after 7 days of rotenone exposure (Fig 3C).

**Fig 4:**
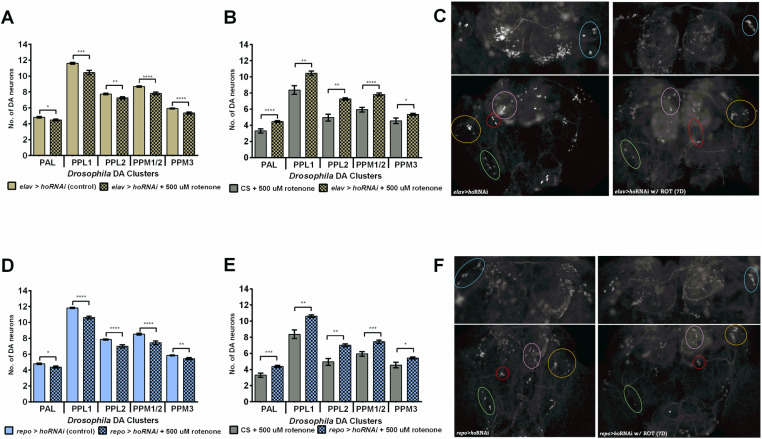
Dopaminergic cell number after rotenone treatment. Flies with chronic *ho* silencing in neurons (A-C) and glia (D-F). Mean ± SEM. Significant differences between rotenone-treated and untreated flies (A,D) as well as between wild-type and transgenic flies (B,E) are marked with asterisks, *p < 0.05, **p < 0.01, ***p < 0.001, ****p < 0.0001. C, F – Images of TH-immunopositive clusters in brains without and with rotenone feeding.

To understand the differences in survival and climbing between the experimental groups, we analyzed the expression of the autophagy gene *Atg5*. Flies with high mortality after rotenone treatment (*elav*>*ho* and *repo*>*ho*) showed decreased levels of *Atg5* gene expression after 3 days of exposure (Fig 3D). On the other hand, *elav*>*hoRNAi* flies, which had similar survival but worse climbing ability after rotenone treatment compared with wild type flies, had the same *Atg5* mRNA level as control (Fig 3E). However, *repo*>*hoRNAi*, which resulted in better survival and climbing results similar to those of CS, had higher mRNA levels of *Atg5* (Fig 4A). Finally, *CncC* overexpression increased *Atg5* expression (Fig 3F). These data suggest that elevated autophagy protects the organism against neurotoxins.

In the next step, we investigated the associations between HO/CncC, rotenone, and apoptosis by checking expression level of *hid*, initiator caspase *Death regulator Nedd2-like caspase* (*Dronc*) and effector caspase *Death caspase-1* (*Dcp-1*) genes. The expression level of selected genes was not affected after 3 days of rotenone treatment in *elav*>*ho* or *repo*>*ho* flies (Fig 3G, 3J, and 3M). In flies with *ho* silencing (*elav*>*hoRNAi*), there were no changes in the *hid* expression level after rotenone treatment, while in the *repo*>*hoRNAi* group, the *hid* mRNA level was greater than that in the control group (Fig 3H). However, *Dronc* mRNA was decreased in *elav>hoRNAi* flies (Fig 3K), while *Dcp-1* was not affected after *ho* silencing (Fig 3N). Surprisingly, *hid, Dronc* and *Dcp-1* expressions were also not affected after 7 days of rotenone exposure in *elav*>*CncC* and *repo*>*CncC* flies (Fig 3I, 3L, and 3O).

To determine the effect of HO/CncC on the immune system we investigated the level of *immune deficiency* (*imd)* expression after rotenone treatment. We observed no changes after rotenone feeding in *elav>ho* and *repo>ho* flies (Fig 3P), while decreased *imd* mRNA level in other groups (Fig 3R and 3S). However, expression of *Relish*, NF-ĸB transcription factor, was unchanged in *elav>hoRNAi* flies after rotenone treatment (Fig 3T–3V).

### Decreased *ho* or increased *CncC* expression in the brain minimizes DA neuron degeneration after chronic rotenone exposure

**Fig 5:**
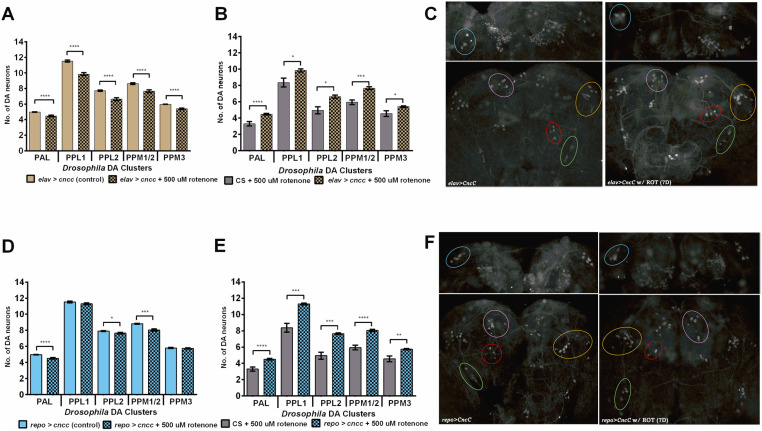
Dopaminergic cell number after rotenone treatment. Flies with chronic *CncC* overexpression in neurons (A-C) and glia (D-F). Mean ± SEM. Significant differences between rotenone-treated and untreated flies (A,D) as well as between wild-type and transgenic flies (B,E) are marked with asterisks, *p < 0.05, **p < 0.01, ***p < 0.001, ****p < 0.0001. C, F – Images of TH-immunopositive clusters in brains without and with rotenone feeding.

Rotenone has been intensively investigated in the case of neurodegeneration of dopaminergic cells. In wild-type flies, 7 days of rotenone exposure caused overall DA neuron loss of ca. 31 ± 4.7% (Fig 4A). The additional control, DMSO, had no effect on neurodegeneration in DA cells after this treatment (Fig S2). Next, flies with silenced *ho* or overexpressed *CncC* that survived after 7 days of rotenone exposure, were examined for DA neuron degeneration and the results were compared with those of the control (Fig 4, Table 1). A significant reduction in the overall percentage of degenerated cells was observed in the rotenone-exposed *elav*>*hoRNAi*, *repo*>*hoRNAi*, *elav*>*CncC*, and *repo*>*CncC* groups. However, we observed that modification of *ho* expression had positive effects: *elav*>*hoRNAi* flies had only approximately 11 ± 2.1%, *repo*>*hoRNAi* - 13 ± 2.9%, and *elav*>*CncC* 14 ± 3.8% missing cells in all five DA neuron clusters (Figs 4A–4F, 5A–5F, Table 1). The most protective treatment for DA neurons was *CncC* overexpression in glia. Those flies had the least DA neuron degeneration of ca. 7 ± 2.9% after exposure to rotenone for 7 days. They had no degenerated cells in the PPL1 and PPM3 DA clusters (Fig 5E, Table 1). After three days of rotenone exposure, the number of cells in *elav*>*ho* and *repo*>*ho* flies did not significantly differ from that of the controls, except in the PPL1 cluster (Fig S3). Wild-type flies did not show any loss of neurons; however, in the case of pan-neuronal *ho* overexpression, we observed neurodegeneration in the PPL1 cluster (Fig S3A), while in the *repo*>*ho* strain, DA neurons were not affected (Fig S3B).

**Table 1:** The number of missing dopaminergic cells. Statistically significant differences between control and rotenone-fed flies are marked in bold (p < 0.05).

	PAL	PPL1	PPL2	PPM1/2	PPM3
*CS*	0.0	0.2	0.1	0.3	0.0
*CS + ROT*	**1.5**	**3.6**	**2.8**	**3.1**	**1.4**
*elav>hoRNAi*	0.2	0.4	0.3	0.3	0.1
*elav>hoRNAi + ROT*	**0.5**	**1.6**	**0.7**	**1.2**	**0.6**
*repo>hoRNAi*	0.2	0.2	0.1	0.5	0.1
*repo>hoRNAi + ROT*	**0.6**	**1.4**	**1.0**	**1.6**	**0.6**
*elav>Cnc*	0.0	0.5	0.3	0.4	0.0
*elav>CncC + ROT*	**0.5**	**2.1**	**1.4**	**1.3**	**0.6**
*repo>CncC*	0.0	0.5	0.1	0.2	0.2
*repo>CncC + ROT*	**0.5**	0.7	**0.4**	**1.0**	0.3
CS SnPPIX (7D)	0.4	0.9	0.8	0.9	0.3
CS SnPPIX + ROT (7D)	0.3	1.2	**1.6**	**1.3**	0.4
CS + curcumin (21D)	0.4	0.5	0	0.3	0.4
CS + curcumin (21D) + ROT (7D)	0.9	**2.4**	**3**	**1.3**	0.9

### Heme oxygenase activity has a negative effect on fly fitness after rotenone exposure

**Fig 6:**
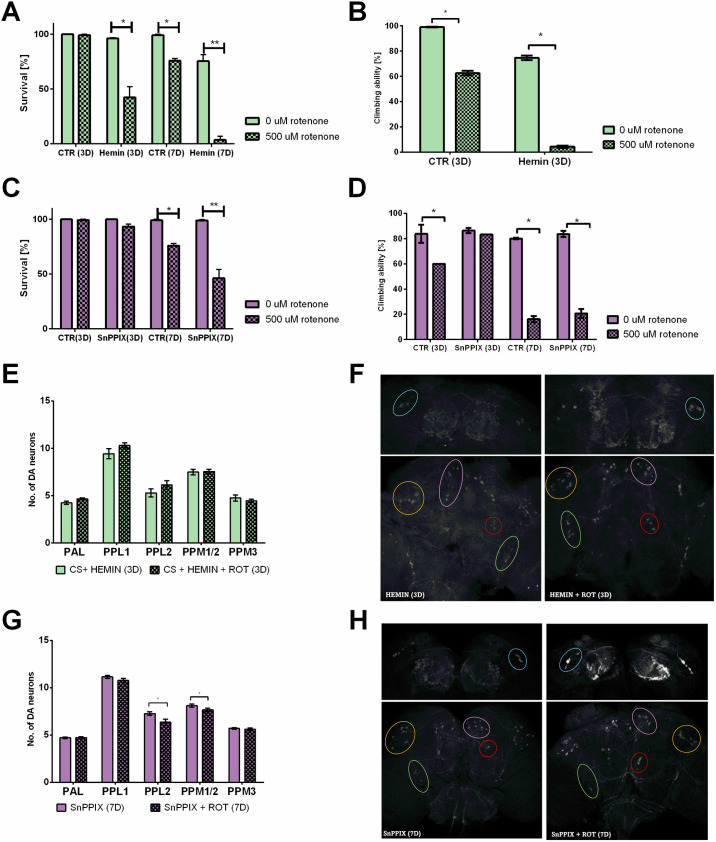
Effects of rotenone treatment on flies fed a HO activator (hemin) or inhibitor (SnPPIX). Because of the high mortality of flies fed hemin and rotenone, experiments were performed after 3 d of feeding (A, B, E, F), while experiments with SnPPIX were performed after 7 d of feeding (C, D, G, H). Mean ± SEM. Statistically significant differences are marked with asterisks: *p < 0.05, **p < 0.01, ***p < 0.001, ****p < 0.0001. F, H – Images of TH-immunopositive clusters in brains without and with rotenone feeding.

In the next step, we examined the effect of hemin (an activator of HO activity) on rotenone-treated flies for 7 days. We observed, similar to *ho* overexpression, very high mortality (Fig 6A). Even after 3 days of hemin/rotenone treatment, survival was reduced to 40% (Fig 6A), and climbing ability was very low (Fig 6B). After feeding with a mixture of SnPPIX (an inhibitor of HO activity) and rotenone, survival did not change after 3 days of treatment (Fig 6C) but decreased to 50% after 7 days (Fig 6C). The climbing ability did not change after 3 days of SnPPIX plus rotenone treatment, and it was only 20% after 7 days (Fig 6D). Three days of hemin/rotenone feeding did not affect dopaminergic cell number (Fig 6E); however, this short duration of rotenone exposure did not induce neurodegeneration in control flies (Fig 6E). However, feeding flies with SnPPIX prevented the neurodegeneration of DA neurons, since after 7 days of rotenone exposure, the number of neurons decreased only in the PPL2 and PPM1/2 clusters (Fig 6F).

### CncC has a neuroprotective effect on dopaminergic cells in flies treated with rotenone

Finally, we investigated the effect of CncC activity on rotenone-treated flies. When flies were fed curcumin (an enhancer of *CncC* expression) and rotenone for 7 days we observed similar lethality and climbing activity as in flies fed with rotenone only. However, our previous studies showed that curcumin feeding is effective after 2 weeks of treatment, when it increases the expression [47] of specific genes. Therefore, we fed the flies curcumin for 2 weeks and then curcumin plus rotenone for the next 7 days. Because the flies exposed to the neurotoxin were already 2 weeks old, we observed a very high mortality rate (80%) (Fig S4A). The experimental flies that survived until the end of the experiment were subjected to brain isolation and TH-positive cell detection. Despite the low number of animals and their age, we observed more dopaminergic cells in the brains of those flies than in young CS treated flies (except for those in the PPL2 cluster) (Fig S4B).

## Discussion

In the present study, we investigated whether Nrf2 or HO can protect dopaminergic neurons after exposure to rotenone, a neurotoxin that induces Parkinson’s disease (PD) symptoms. We used *Drosophila melanogaster* as a model to examine the effects of chronic *CncC* (Nrf2 in mammals) and *ho* overexpression and silencing, as well as chemical modifications of HO and Nrf2 activity, on flies treated with rotenone.

Upregulation of Nrf2 expression suppresses PD-like phenotypes in *Drosophila* [57,58]. Wang *et al*. [57] showed that selective overexpression of *CncC*, particularly in DA neurons, decreases ROS levels and attenuates DA neuron degeneration in transgenic flies expressing mutated α-synuclein (A53T). Guo *et al*. [58] highlighted the importance of glial cells (particularly astrocytes) in the activation of Nrf2 in mammals and of CncC in flies to inhibit neurodegeneration induced by neurotoxins. Similarly, Nrf2 overexpression restricted to astrocytes rendered mice resistant to neurotoxins [59,60] and delayed or minimized the risk of neurodegeneration in mutants with genetically elevated α-synuclein [61]. These reports suggest that neuronal Nrf2 is not explicitly required to prevent neuronal death and that astrocytes are key mediators of Nrf2 signaling. Our data support these findings, as we observed that the flies with overexpression of *CncC* in glia were the least affected by chronic rotenone exposure, since they had the highest survival percentage, the lowest percentage of climbing deficit, and the lowest risk of DA degeneration. In addition, they exhibited normal levels of apoptotic gene expression and decreased immune response after rotenone treatment, which suggests that CncC can inhibit apoptosis and inflammation under stress conditions. In turn, flies in which the expression of *CncC* was partially suppressed showed low resistance to a sublethal dose of rotenone, due to high ROS concentration in the head. These findings indicated that Nrf2 in *Drosophila* is responsible for neuroprotection against rotenone-induced toxicity.

It is also essential to emphasize that CncC acts as an upstream regulator of autophagy in protecting against neurodegeneration, as suggested by other authors [58], which was also shown in our study. Autophagy is involved in a wide range of processes, including intracellular clearance, suppression of tumor formation, aging, cell death and survival, and immune responses [62]. It is widely considered a primary protective mechanism that maintains cellular homeostasis in response to stress. Dysregulation of the autophagic pathway has been detected in the brains of PD patients and animal models of PD, including *Drosophila* models. Additionally, the proteins linked to autosomal dominant PD were reported to be involved in the autophagy pathway, including mitophagy, in recessive PD [63,64]. The autophagy pathway seems to play an important role in the cellular dysfunctions associated with PD, which explains the abundance of recent experimental and clinical approaches critically directed at identifying potential treatments or therapies that enhance autophagy.

Guo *et al*. [58] reported the induction of HO in flies with *CncC* overexpression after rotenone treatment. This finding is in contrast with our results after using 500 µM rotenone. However, in our study, we used higher concentrations of rotenone than those used by Guo *et al*., because, according to Coulom & Birken [56], 125 µM rotenone induces 20% of overall DA neuron degeneration which increases, along with locomotor impairments, with higher doses up to the maximum sublethal limit of 700 µM [56]. Low concentrations can activate a “hormetic” dose response [65], which is also mediated by Nrf2 [66]. We suggest that hormesis has an impact on data interpretation, especially when experiments involve exposure to neurotoxins.

We previously documented that our flies with *CncC* overexpression had upregulated *ho* mRNA levels compared to the parental controls [49]. Therefore, we assumed that the lack of changes in *ho* expression in flies overexpressing *CncC* after chronic rotenone exposure may imply that *ho* mRNA expression is still high but at the same level as that in untreated flies. Additionally, compared with their GAL4 and UAS controls, flies with continuous *ho* overexpression exhibit high expression of apoptotic genes under normal conditions [48]. This may explain the low percentage (8–22%) of surviving a sublethal dose of rotenone for 7 days in flies overexpressing *ho*, which also independently stimulates the activation of apoptosis. *ho* overexpression is not beneficial for flies, even in the absence of additional stressors [49]. We also employed a short exposure time to rotenone for 3 days to show that autophagy is a pro-survival mechanism, but with *ho* overexpression in the brain, survival gradually decreased. Although the expression level of the apoptosis gene *hid* was not changed by rotenone, it was significantly greater in young adults with *ho* overexpression than in the GAL4 and UAS parental controls [48]. Similar results were observed for *ho* silencing in our experiments, which may explain the greater percentage of climbing deficits in flies and the greater percentage of degenerated DA neurons in these flies than in those with *CncC* overexpression in glia. However, in comparison to flies with *ho* overexpression, they survived better after chronic exposure to rotenone, and they had fewer degenerated DA neurons. Surprisingly, *ho* silencing in glia facilitated the induction of autophagy, which probably helped them to have better resistance to rotenone toxicity than did *ho* silencing in neurons. Previously, we also showed that under normal conditions, changing *ho* expression levels in glia did not affect apoptosis or autophagy [47]. Here, we confirmed that both processes can increase only under oxidative challenge. Partially decreasing *ho* expression is also a potential therapeutic approach against neurodegeneration induced by oxidative stress and mitochondrial impairment, which has also been reported by other authors [54,67–76].

Our results highlight the importance of heme oxygenase and Nrf2 in neurodegenerative processes under environmental stress. This approach provides new opportunities for studies on possible protective or risk agents. HO and Nrf2 have chemical activators and inhibitors that can be delivered with food as supplements. There are also natural bioactive substances, such as curcumin, which could be used to prevent the consequences of exposure to neurotoxins. Our results showed that manipulations of HO activity and its level have negative effects on the organism. All aspects tested—survival, climbing ability, and dopaminergic cell viability—were impaired after treatment with hemin, an activator of HO. On the other hand, feeding with SnPPIX, an HO inhibitor, increased climbing ability and dopaminergic cell survival; however, it increased mortality. This effect could be explained by the hormesis effect. Lower HO activity increases ROS levels, which stimulate protective mechanisms in the body. On the other hand, above the critical level, ROS are toxic. Moreover, hyperactivation of protective processes requires more energy, which increases the risk of death. It is also possible that different concentration and duration of exposure may have a positive effect without affecting survival. These results are important, because they indicate that supplements need to be carefully examined for their longtime action and side effects. Notably, the expression of the *ho*, autophagy, and apoptosis genes is under the control of the circadian clock and shows daily oscillations [43,45,77–79].

The most promising results were obtained for curcumin treatment. This agent has antioxidative properties and is commonly used as a food colorant and supplement. Curcumin is known as an Nrf2 activator, and indeed, we showed in our previous studies that two weeks of curcumin treatment activates CncC in the fly’s head [47]. It has also been shown that curcumin protects against low concentrations of rotenone, which leads to cell death, in *in vitro* and *in vivo* experiments through the inhibition of caspase-3 and caspase-9 induction [80]. Our data confirmed these findings because at higher rotenone concentrations, we observed less degenerated dopaminergic cells, even in 3-week-old flies. These findings are very promising because they show that curcumin has a protective effect on rotenone-exposed brain.

In conclusion, Nrf2 activation in glial cells is a potential neuroprotective mechanism against brain dysfunctions associated with PD. However, it should include restricting HO activity, as it may increase apoptosis. For instance, the factors involved in the selective binding of Nrf2 to the antioxidant response element (ARE), which is found in the promoter region of several genes encoding detoxification enzymes and cytoprotective proteins, should be investigated. Other antioxidant enzymes affected by Nrf2 should also be explored because they can synergistically affect autophagy rather than HO to improve its effectiveness against rotenone-induced toxicity.

## Materials and methods

### Experimental animals

The following strains of *D. melanogaster* were used: wild-type Canton S (CS); *elav*-Gal4 (pan-neuronal cell marker); *repo*-Gal4 (pan-glial cell marker); UAS-*hoRNAi* (kind gift from Taketani, Japan); UAS-*ho* (kind gift from Taketani, Japan); UAS-*CncC* (BDSC no. 95246); and UAS-*CncCRNAi* (BDSC no. 25984).

The GAL4/UAS system was used to generate flies with *ho* overexpression or silencing in all neurons (*elav*>*ho* or *elav*>*hoRNAi*) or in all glia (*repo*>*ho* or *repo*>*hoRNAi*) as well as for *CncC* overexpression or silencing (all neurons: *elav*>*CncC* or *elav*>*CncCRNAi*, all glia: *repo*>*CncC* or *repo*>C*ncCRNAi*). The efficiency of silencing/overexpression was checked using qPCR (Fig S5). Adult males of each genotype (including CS) were transferred for 3 or 7 d to vials containing cotton soaked with either DMSO (control) or 500 μM rotenone (Sigma) dissolved in DMSO (experimental flies), which was mixed in 10% sucrose. For the experiments, we used rotenone concentrations according to Coulom & Birman [56]. The control group was fed standard yeast-cornmeal-agar media for comparison with the group exposed to DMSO in 10% sucrose.

To confirm the effect of *ho* overexpression and silencing, we performed experiments using wild-type CantonS flies fed 500 μM rotenone mixed with 100 μM hemin (HO activator, Calbiochem) or 100 μM tin protoporphyrin IX or SnPPIX (HO inhibitor, Frontier Scientific) for 3 or 7 d, respectively.

To confirm the effect of *CncC* overexpression, we used wild-type CantonS flies fed curcumin (1 mg/mL in culture medium, EMD Millipore Corp.) for 14 d, followed by 7 d of treatment with curcumin mixed with 500 μM rotenone.

All flies were maintained under 12 h of light followed by 12 h of darkness (LD12:12).

### Survival and climbing assays

After feeding with rotenone or DMSO (control) for 3 or 7 d, the number of live males of each genotype was counted. Flies from the experimental and control groups that survived exposure to rotenone and DMSO, respectively, were then transferred to an empty vial, and after a short recovery, they were gently tapped to the bottom of the vial. After 15 s, individuals who climbed vertically beyond the 5-cm marked line were counted. The climbing assay was carried out at ZT1 under dim red light conditions. Both assays were repeated at least three times with 30 males per repetition per genotype.

### RNA isolation and gene expression

Males of each genotype were collected at ZT1 or ZT16 (Zeitgeber Time; ZT0 indicates lights-on, and ZT12 indicates lights-off). Heads were isolated and subjected to total RNA isolation using TriReagent (MRC, Inc.) according to the manufacturer’s protocol. The RNA quality and quantity were assessed using a Nanodrop 2000 (Thermo Fisher Scientific). cDNA was synthesized using 500 ng of total RNA and a High Capacity cDNA Reverse Transcription Kit (Thermo Fisher Scientific) with random primers according to the manufacturer’s instructions. Gene expression was examined using the StepOnePlus Real-Time PCR System and SYBR Fast ABI Prism (KAPA Biosystems) in the presence of specific primer sequences (the specificity was controlled with Primer–BLAST and gel electrophoresis) for the target genes, which are listed in Table 2. This entire procedure was followed in experiments analyzing gene expression levels, which were repeated at least three times per genotype with approximately 20 fly heads for each replicate.

**Table 2:** Primer sequences of the genes used in this study.

Gene		Sequence	Accession Number
*Ho*	F:	5’-ACCATTTGCCCGCCGGGATG-3’	CG14716
	R:	5’-AGTGCGACGGCCAGCTTCCT-3’	
*RpL32*	F:	5’-TATGCTAAGCTGTCGCACAAATG-3’	CG7939
	R:	5’-AGCACGTGTATAAAAAGTGCCA-3’	
*Hid*	F:	5’-CATCCATGGCCACATCAGT-3’	CG5123
	R:	5’-TTACACGTCTCCTGCGCTTT-3’	
*Atg5*	F:	5’-GACATGCTCGTCAAGCTCAA-3’	CG1643
	R:	5’-TCCATTAGCCTCCGATTGAC-3	
*Relish*	F:	GGTGATAGTGCCCTGCATGT	CG11992
	R:	CCATACCCAGCAAAGGTCGT	
*Dcp-1*	F:	GGAAAATCGGGGCAGCTTTAT	CG5370
	R:	CATTCGAGCCACAAACTTGTTG	
*Dronc*	F:	CCTTTATCTCGCTAAACGAACGG	CG8091
	R:	AGCTTGCTAACGCAGGGTC	
*imd*	F:	TCAGCGACCCAAACTACAATTC	CG5576
	R:	TTGTCTGGACGTTACTGAGAGT	

### Brain immunostaining

Heads were fixed at ZT1 in 4% paraformaldehyde (PFA) in phosphate-buffered saline (PBS) for 1 h at RT. Then, the brains were isolated and washed in PBS for 10 min and three times in 0.2% PBS with Triton X-100 (PBST) for 10 min each. Next, they were incubated in 5% normal goat serum (NGS) in 0.2% PBST for 45 min at RT. Subsequently, the brains were incubated for 3 days at 4 °C with mouse primary anti-tyrosine hydroxylase antibodies (1:1000, ImmunoStar). Thereafter, the brains were washed three times in 0.2% PBST for 10 min each and incubated for 2 h at RT with secondary goat anti-mouse Cy3-conjugated antibodies (1:250, Abcam). Finally, the brains were washed three times in 0.2% PBST and once in PBS for 10 min each before being mounted in Vectashield medium (Vector). Z-stacks of whole-mount brains were obtained with a Zeiss LSM780 laser scanning microscope. Direct counting of DA neurons was performed in each of the five DA clusters, namely, PAL (protocerebral anterior lateral), PPL1 (posterior inferior lateral protocerebrum), PPL2 (posterior lateral protocerebum), PPM1/2 (posterior superiormedial protocerebrum and posterior inferomedial protocerebrum), and PPM3 (superior posterior slope) [81], using ImageJ software. This experiment was repeated three times per genotype with approximately 10 heads for each replicate.

### ROS measurement

DCFH-DA (Invitrogen) was used to measure reactive oxygen species (ROS) levels. Flies, 20 per sample, were decapitated at the same time of the day. They were manually homogenized in 20 µM DCFH-DA in Schneider’s medium (SM) and incubated at 25 °C for one hour. Homogenate was centrifuged for 5 min at 800 rcf, pellet was washed twice in fresh medium, and then resuspended in 100 µl of SM. Samples were sonicated and centrifuged for 5 min at 14 000 rcf. The absorbance of the samples was measured by a spectrophotometer at 488 nm. Results were normalized to the amount of protein in the sample (mg/ml).

### Statistical analysis

All the data were examined for normal distribution, and statistical tests were chosen accordingly. The nonparametric Mann‒Whitney test was used to assess differences in percent survival, percent climbing, and gene expression between the experimental and control groups. The results obtained for DA degeneration after feeding with or without rotenone were analyzed using an unpaired t test with Welch’s correction. Statistical analyses were performed with GraphPad Prism 7.05 software.

## Supporting information

S1 Fig Changes in the levels of ROS in heads of flies treated with rotenone. (TIF)

S2 Fig The number of dopaminergic cells in control flies fed with normal diet and DMSO + sucrose. (TIF)

S3 Fig The number of Th-immunopositive cells after 3 days of rotenone treatment of wild type Canton S, pan-neuronal *ho* overexpression (*elav>ho*) and pan-glial *ho* overexpression (*repo>ho*) flies. (TIF)

S4 Fig The effect of feeding with curcumin (21 days) and rotenone (7 days) on survival and the number of dopaminergic cells. (TIF)

S5 Fig The expression level of *ho* and *CncC* after silencing or overexpression in neurons or glia. (TIF)
