# Supplementary figures and images for: The neuroprotective role of CncC in a Drosophila model of Parkinson’s disease
Source: PLoS One. 2025 May 13;20(5):e0322640. doi: 10.1371/journal.pone.0322640 (PMC12074349; doi:10.1371/journal.pone.0322640)

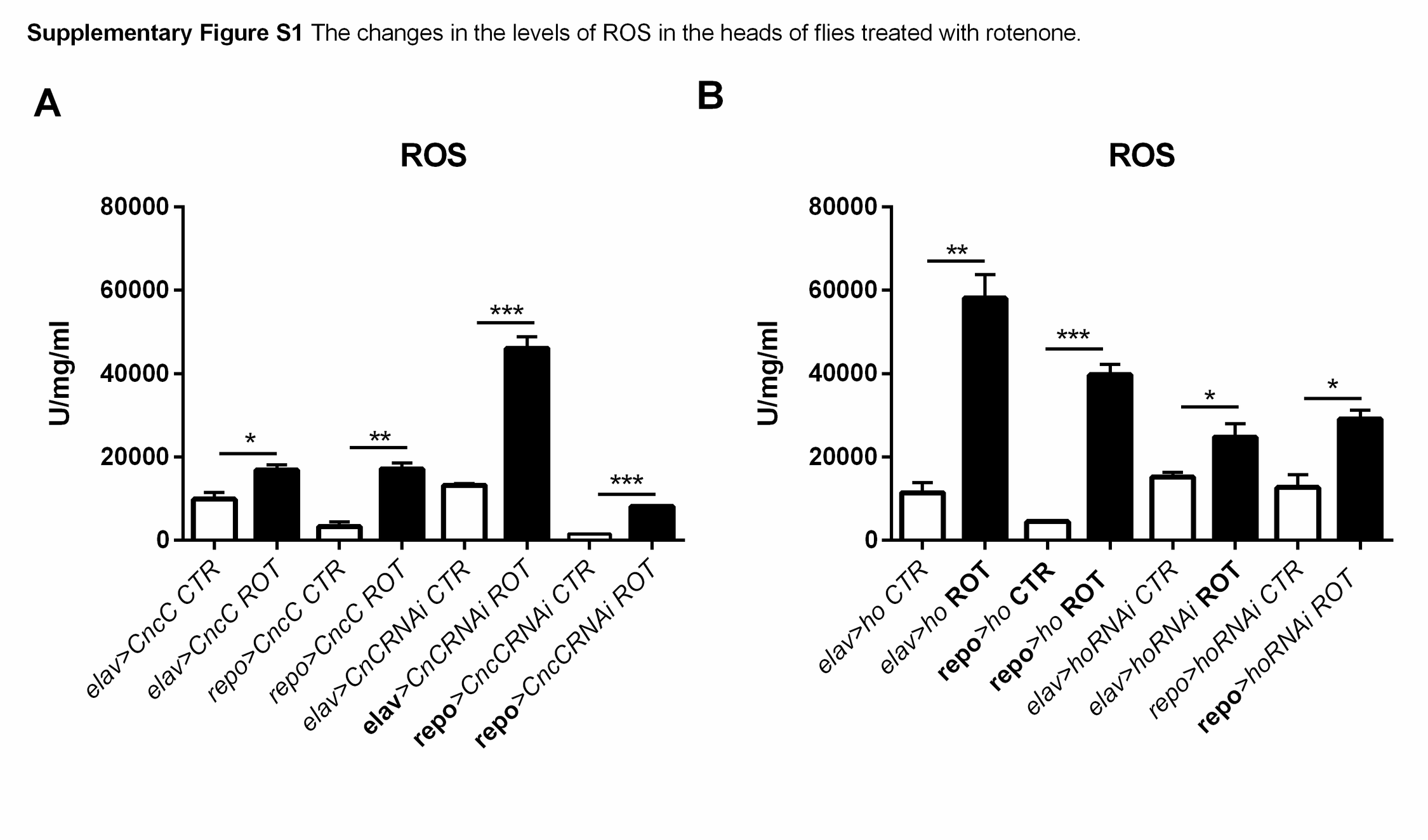

Supplement: S1 Fig — (TIF) [file pone.0322640.s001.tif]

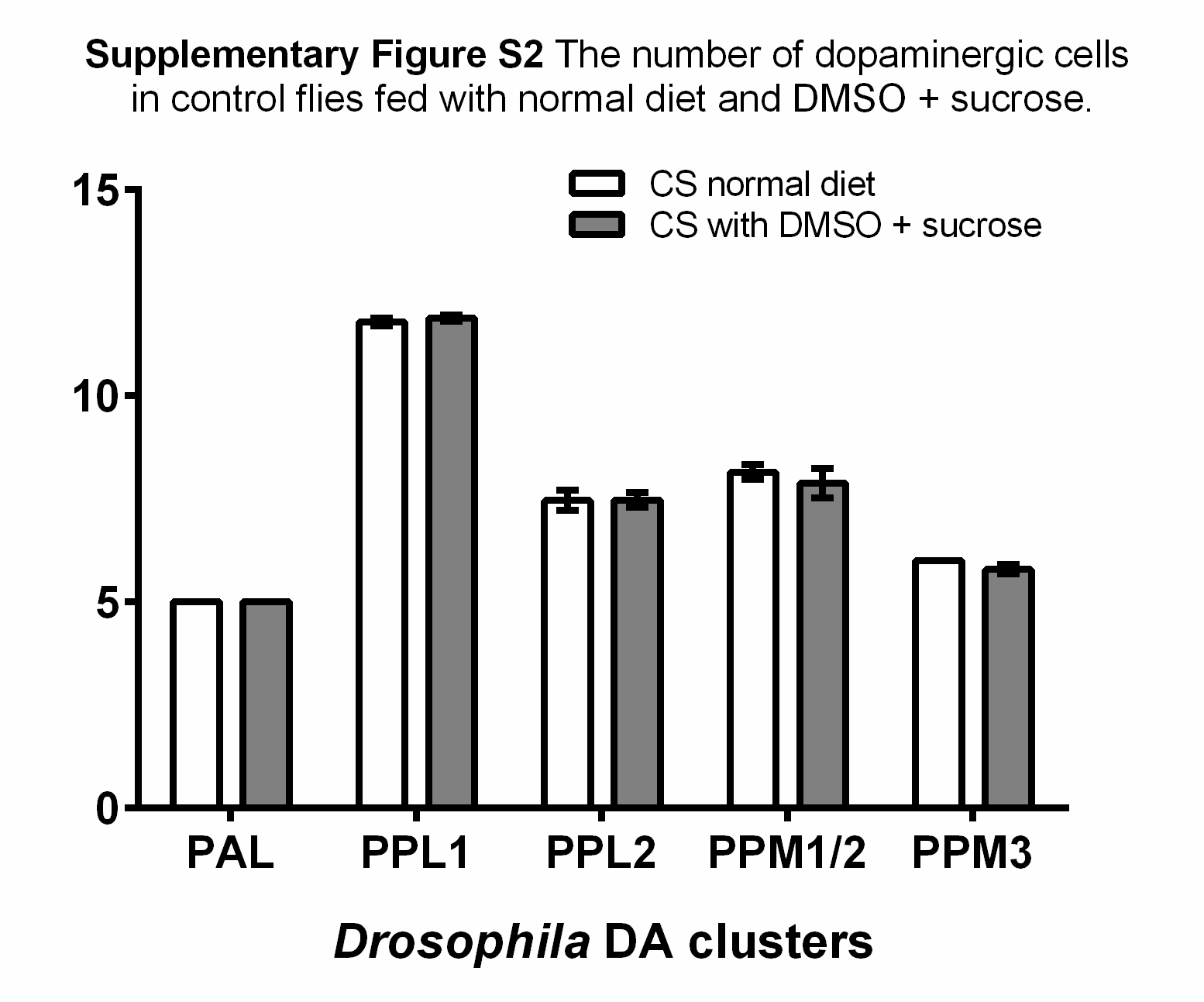

Supplement: S2 Fig — (TIF) [file pone.0322640.s002.tif]

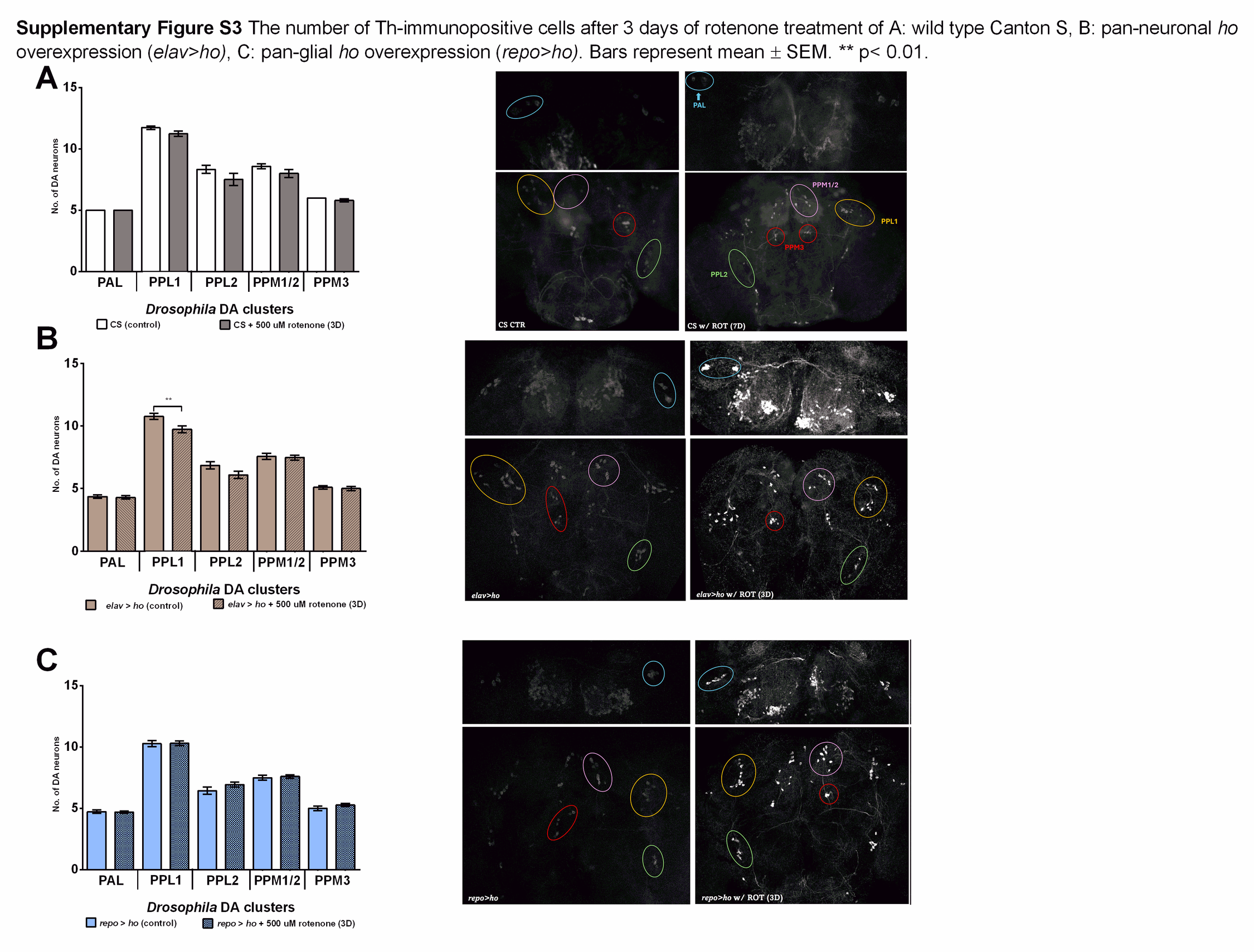

Supplement: S3 Fig — (TIF) [file pone.0322640.s003.tif]

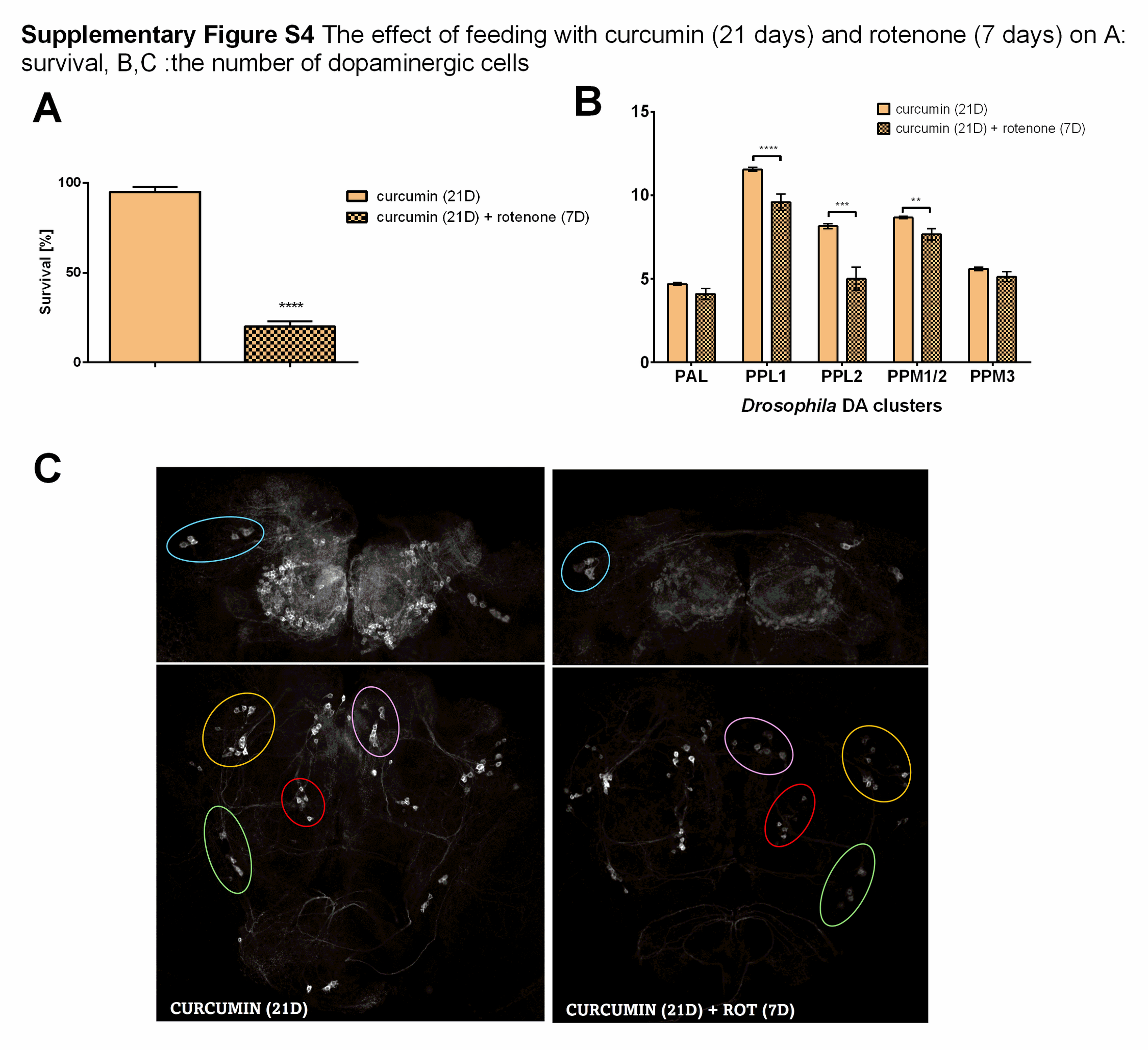

Supplement: S4 Fig — (TIF) [file pone.0322640.s004.tif]

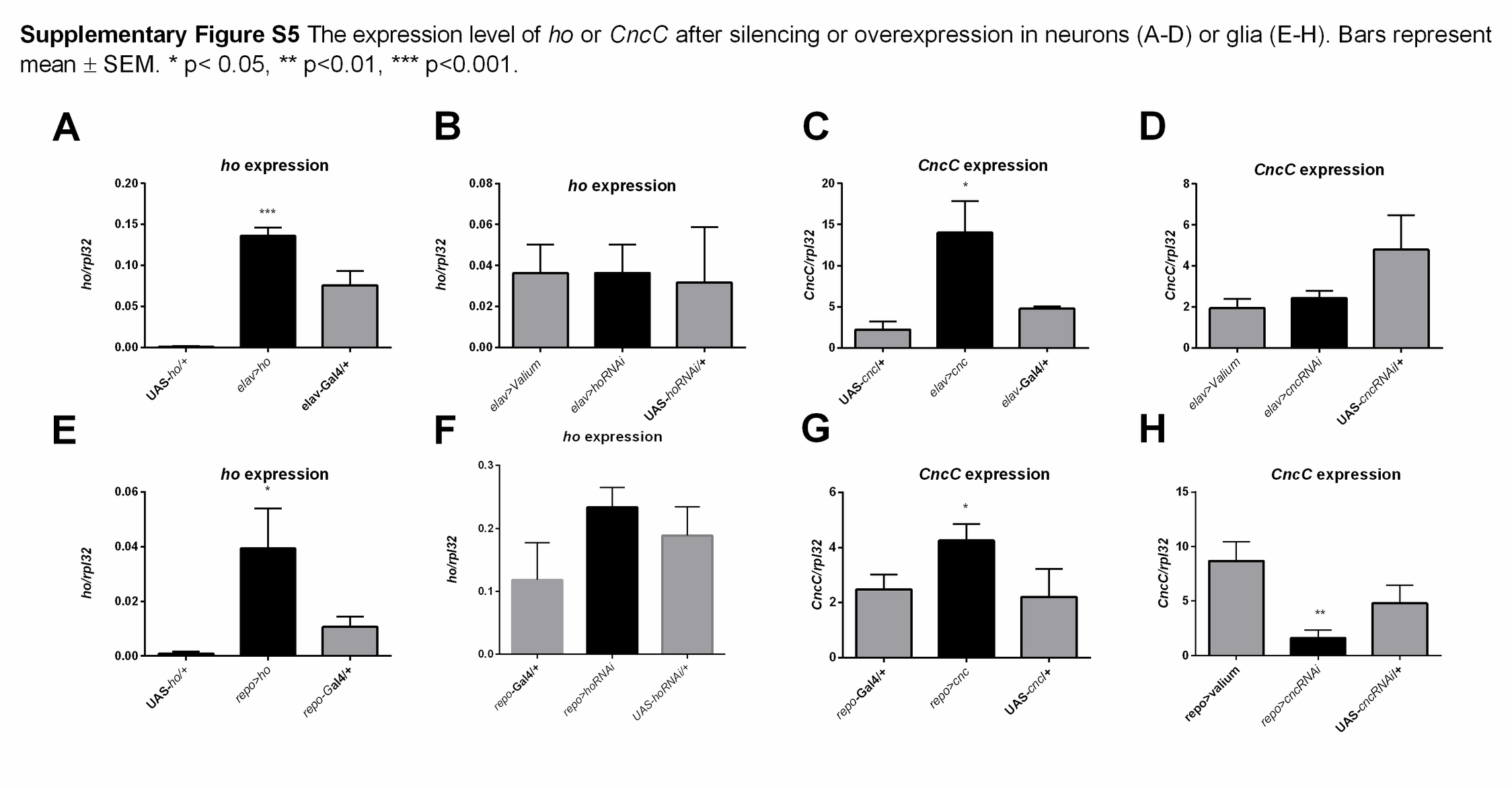

Supplement: S5 Fig — (TIF) [file pone.0322640.s005.tif]
